# Mpox vaccine acceptance among healthcare workers: a systematic review and meta-analysis

**DOI:** 10.1186/s12889-023-17186-2

**Published:** 2024-01-02

**Authors:** Ammar Mektebi, Mohamed Elsaid, Tularam Yadav, Fatima Abdallh, Mohamad Assker, Abdelmonem Siddiq, Reem Sayad, Motaz Saifi, Ramadan Abdelmoez Farahat

**Affiliations:** 1 Faculty of Medicine, Kutahya Health Sciences University, Kutahya, Turkey; 2 Medical Research Platform, Cairo, Egypt; 3 German-Syrian Research Society e.V., Frankfurt, Germany; 4 Faculty of Medicine, 6Th of October, Misr University for Science and Technology, Giza, Egypt; 5 Faculty of Medicine, Jinnah Postgraduate Medical Centre (JPMC), Karachi, Pakistan; 6 Faculty of Medicine, Hashemite University, Zarqa City, Jordan; 7 Faculty of Medicine, University of Sharjah, Sharjah, UAE; 8 Faculty of Pharmacy, Mansoura University, Mansoura, 35516 Egypt; 9 Faculty of Medicine, Assiut University, Assiut, Egypt; 10 Department of Medicine, Medicine & Health Science, An-Najah National University, Nablus, Palestine; 11 Faculty of Medicine, Kafrelsheikh University, Kafrelsheikh, Egypt

**Keywords:** Mpox, Vaccine, Vaccine acceptance, Vaccine hesitancy, Healthcare workers, Meta-Analysis

## Abstract

**Introduction:**

Mpox is a zoonotic viral disease that emerged in May 2022 and has since shown a high prevalence in non-mpox-endemic areas, resulting in an outbreak that caused more than 84,000 cases in 110 countries around the globe. Several vaccines are available to prevent the disease, and multiple studies have been conducted to assess the attitudes of different populations toward receiving the mpox vaccine. This study systematically reviews all the studies conducted on mpox vaccine acceptance/hesitancy among healthcare workers.

**Methods:**

A systematic literature search was conducted through four electronic databases, including PubMed, Scopus, Web of Science, and Google Scholar, up to March 2023. Studies that described mpox vaccine acceptance/hesitancy among healthcare workers were included, and the data were extracted using a uniform extraction sheet. Following the extraction, the meta-analysis included ten studies with 7322 healthcare workers. Three researchers independently assessed the risk of bias in the included study using the Newcastle–Ottawa Scale (NOS).

**Results:**

Ten studies were included in the review. This review indicates that the prevalence of mpox vaccine acceptance was 58.5%, and the prevalence of mpox vaccine hesitancy was 41.5%. There was a higher prevalence of acceptance in countries located in Asian and African areas compared to those in North America and Europe, estimated at 68% and 44.3%, respectively. Among the studies conducted solely among physicians, there was a high prevalence of mpox vaccine acceptance, at 77.1%, compared to 49% in studies that included all healthcare workers.

**Conclusion:**

There is a significant variation in the prevalence of mpox vaccine acceptance among different populations. Further research is needed to identify the factors that contribute to this variation and to develop interventions to increase vaccine acceptance. In addition, it is important to promote research on mpox vaccine acceptance and hesitancy among healthcare workers in countries where data is limited. This research will help policymakers develop effective policies to increase acceptance and reduce the disease burden.

**Supplementary Information:**

The online version contains supplementary material available at 10.1186/s12889-023-17186-2.

## Introduction

While the world has COVID-19 pandemic end in sight, a multi-country outbreak has emerged and been declared a public health emergency of international concern (PHEIC) by the WHO, indicating that mpox is one step away from becoming a pandemic [1–3]. The outbreak was caused by a double-stranded DNA virus called Monkeypox virus (MPXV), which belongs to the Poxviridae family, the same family as the variola virus, the causative agent of smallpox [4, 5]. In addition to other members of the family Poxviridae that harm birds, livestock, cervids, c rocodiles, rabbits, and insects, members of the genus Orthopoxvirus cause sickness in both humans and animals. It spread via respiratory secretions, skin contact or breached mucosal wounds, and exposure to contaminated goods [6]. Also, it is important to highlight that this is not the first time MPXV has made a breakout; in 2003, it was reported for the first time outside its endemic area, Africa [7, 8].

Until 9^th^ August 2023, the WHO reported more than 89,000 cases with 152 deaths among 113 countries around the globe that had never previously reported MPXV cases. The majority of affected populations were male (96.3%) showing a median age of thirty-four years. Moreover, the Western Pacific, European, and American regions have seen a rise in reported cases during the past two weeks, with a clinical presentation quite similar to smallpox (since they share structural similarities) [9, 10]. Patients almost always had a skin rash in addition to various symptoms like fever, pruritis, and lymphadenopathy, which were the most commonly reported, with the possibility of being hospitalized or, less likely, ending in fatal outcomes such as respiratory infection, sepsis, or encephalitis and expected incubation period vary from five days to twenty-one days [11].

After the first mpox outbreak, global efforts were made to control and prevent the virus from spreading, primarily through vaccination. JYNNEOS vaccine -a replication-deficient vaccinia virus vaccine- is currently being used for this purpose and was approved by the Food and Drug Administration (FDA) after being proven to be the safest available substitute for the previously used vaccine (ACAM2000), which was linked to reports of dangerous side effects like myopericarditis (most commonly) or even death of unvaccinated people due to contact with vaccinated ones [12–15]. The most frequently reported side effects of JYNNEOS were reactions at the injection site, tightness in the throat, headache, myalgia, chills, and nausea, which were milder and easier to manage compared to ACAM 2000s [15].

Being a front liner in context of prevention of spread of infectious diseases, the Health care professionals (HCPs) must have considerable knowledge (including mechanism of action, effectiveness, benefits, and possible short-term or long-term adverse effects) and understandings about Mpox vaccine for the acceptance among themselves and also educating the society for the establishment of positive attitude and trust. It plays role in substantial reduction of hesitancy to receive the vaccines where available, which WHO identified as one of worrisome challenges in mass vaccination.

During the outbreak, many studies on various populations were conducted to assess perceptions and attitudes toward the mpox vaccine and highlight the vaccine’s acceptance. Studies on healthcare providers were established to check their acceptance of the vaccine. The results showed a wide variation in answers because they depended on multiple variables, like age group, hospital level, and belief in the necessity of the vaccine itself [16–19]. In addition, based on our knowledge, there was no systematic review talking about the overall acceptance of healthcare workers regarding the mpox vaccine. So, from this point, we saw the need to conduct this systematic review to fill this gap and shed light on this crucial topic because healthcare providers are on the front lines of dealing with this outbreak, and their perspectives on it should be highlighted and taken into account when planning for the coming days. Thus, we conducted this review to assess the prevalence of mpox vaccine acceptance among healthcare workers.

## Methodology

This paper presents a systematic review and meta-analysis that follows the Preferred Reporting Items for Systematic Reviews and Meta-Analyses (PRISMA) guidelines to ensure a rigorous and transparent methodology (Figure S1) [20]. The study aims to consolidate the existing evidence on mpox vaccine acceptance/hesitancy among healthcare workers, providing robust and comprehensive conclusions that can inform decision-makers and researchers. The methodology section details the steps to identify, assess, and precisely synthesize the relevant literature. Our protocol has been registered in PROSPERO with CRD42023394597.

### Search strategy

Our search was centred on the PICO criteria (Table 1) with the research question, “What is the prevalence of mpox vaccine acceptance among helathcare workers?”. A comprehensive search strategy was conducted to identify relevant studies, targeting four electronic databases: PubMed, Scopus, Web of Science, and Google Scholar up to March 11th, 2023. The search strategy utilized Medical Subject Headings (MeSH) terms, Emtree terms, and relevant keywords to capture all relevant literature. The following keywords were adapted to be used either alone or in combination to conduct the literature search: “healthcare worker”, “hcw”, “healthcare professional”, “clinician”, “nurse”, “midwife”, “willingness”, “acceptance”, “hesitancy”, “vaccine”, and “mpox”. Besides, many publications were identified from reference lists of relevant articles using the “Snowball Method. The search strategies for each database can be found in Table S1 in the Supplementary Materials.

**Table 1:** Inclusion and exclusion criteria

	Inclusion	Exclusion
Participants	Healthcare workers	Other populations
Intervention	Mpox vaccine acceptance	
Comparator(s)/control	Mpox vaccine refusal	
Main outcome	Prevalence of mpox vaccination acceptance among healthcare workers	
	Sub-group analysis according to	
Study Designs	Prevalence studies, crosssectional studies, surveys	Qualitative, policy, opinion, case studies, case-reports, case series, cohort studies, casecontrol studies
	Geography-Global level Date of Search- Publish till March 11th 2023 Human studies	Non-human studies

### Study selection

Two independent reviewers (TY and FA) conducted a systematic screening process that evaluated abstracts obtained from literature searches. Full-text versions of articles that met the inclusion criteria were retained for further assessment. The authors established an agreement on the inclusion and exclusion criteria, which are available in Table 1. Articles had to be observational studies, involve healthcare workers, and evaluate mpox vaccine acceptance. Studies were excluded if they were reviews, randomized controlled trials (RCTs), non-randomized controlled trials, comments, case reports, or case series; lacked comparison data; or included a different population. No other significant restrictions were imposed to ensure that all relevant articles were included in the analysis. Any discrepancies between the reviewers during the selection process have been resolved through discussion or consultation with a third independent reviewer if necessary (AS and AM).

### Data extraction

Two reviewers (TY and AS) independently extracted the data using a standardized data extraction form. The extracted data included study characteristics such as authors, publication year, country, and participant characteristics such as sample size, age, and gender. Details of the data collection method, such as date, duration, and reported answers, were also extracted, along with outcome measures such as effect size, confidence intervals, and p-values. Any discrepancies in data extraction were resolved through discussion or consultation with a third reviewer (AM) if needed.

### Quality assessment

The methodological quality of the included studies has been assessed using the Newcastle–Ottawa Scale (NOS). The possible quality assessment score ranges from zero to ten points, with a high score indicating good study quality [21]. Two independent reviewers (MA and FA)evaluated the risk of bias, and any disagreements were resolved through discussion or consultation with a third reviewer if necessary (AM).

### Data synthesis

All statistical analyses were done by R Studio for Windows [22]. A variety of responses signifying vaccine acceptance, including participants expressing willingness by agreeing or confirming their agreement with a "yes" response to a confirmation question as outlined in the characteristics table (Table 2), were collectively categorized as acceptance within the statistical analysis. Due to relatively high heterogeneity between studies, we used a random effects model with a 95% confidence interval for the meta-analyses. To identify sources of heterogeneity, we considered performing subgroup analysis based on geographical location (Americas and Europe vs. Asia and Africa), profession (physicians vs. all HCWs), as well as data collection dates and study quality. However, we could not perform subgroup analysis for the data collection date and quality of studies due to the limited variability of these variables. We conducted a leave-one-out sensitivity analysis to assess the impact of each study on the overall mpox vaccine acceptance among HCWs. We inspected funnel plots and assessed Egger’s test (with a *p*-value < 0.05 representing publication bias) to assess the publication bias [23]. There was limited data and high heterogeneity in investigating predictors for mpox vaccine uptake among HCWs. Thus, a meta-analysis was not conducted, but instead, the percentage of studies with positive, negative, or no significant relationships (*p*-value < 0.05) was presented to demonstrate the impact of predictors. Table S3 in the Supplementary Materials provides effect measures between predictors and vaccine acceptance, such as odds ratios and confidence intervals. These measures can be used to gain insights into the relationship between predictors and vaccine acceptance. In cases where the odds ratio was not calculated or there was insufficient data, the percentage ratio was used instead. This was calculated by dividing the prevalence percentages provided in the papers.

**Table 2:** Characteristics of the included studies

Study ID	Country	Physicians N(%)	Female N(%)	Study Design/Survey Type	Data Collection Date	Sample Size	Response Recorded As Acceptance Among HCW	Prevalence Of Vaccine Acceptance	Response Recorded As MPOX Vaccination Refusal Among HCW
Bates et al. 2022 [29]	USA	197 (100)	69 (35.0)	cross-sectional study	September 2–11, 2022	197	Agreeing with / strongly agreeing with receiving the vaccine	96(48,3)	strongly disagreeing with / disagreeing with / being neutral
Hong et al. 2022 [15]	China	406 (39.34)	766 (74.22)	cross-sectional study	May 30th,2022—August 1st, 2022	1032	strongly willing / probably willing	930(90,12)	probably unwilling / strongly unwilling
Alarifi et al. 2022 [28]	Saudi Arabia	297 (40)	378 (50.87)	cross-sectional study	September 13th, 2022—November 13th, 2022	743	Yes	392(52,7)	No
Swed et al. 2022 [27]	(Egypt, Saudi Arabia, Yemen, Syria, Libya, Algeria, Tunisia, Iraq, Palestine, Jordan, and Sudan)	1183 (30.7)	2171 (56.3)	cross-sectional study	2 August to 28 December 2022	3856	Yes	2102(54,5)	No
Ricco et al. 2022 [25]	Italy	163(100)	106 (65.0)	cross-sectional study	24 May 2022 and 31 May 2022	163	Favorable / Highly favorable	105(64,4)	NR
Harpan 2020 [23]	Indonesia	407 (100)	279 (68.6)	observational cross-sectional study / validated questionnaire	May 25, 2019, and July 25, 2019	407	NR	381(93,6)	NR
Salim 2022 [24]	Indonesia	75 (100)	26 (34.7)	descriptive cross-sectional study	August 2nd and August 5th, 2022	75	Yes	58(77,3)	No
Riad 2022 [17]	Czech Republic	36 (10.6%)	138 (90.2)	analytical cross-sectional study	22-Sep	341	NR	30(8,8)	Rejection/Hesitancy
A gagneuxbrunon 2022 [26]	France and Belgium		260 (65.5)	an anonymous online survey	15th June 2022 to 8th August 2022	397	NR	220(55,4)	NR
Lounis et al. 2023 [30]	Algeria	45(40.5)	78(70.3)	A cross-sectional Web-based survey	28 June and 18 September 2022	111	NR	43(38,7%)	NR

## Results

### Article identification and selection

**Fig. 1:**
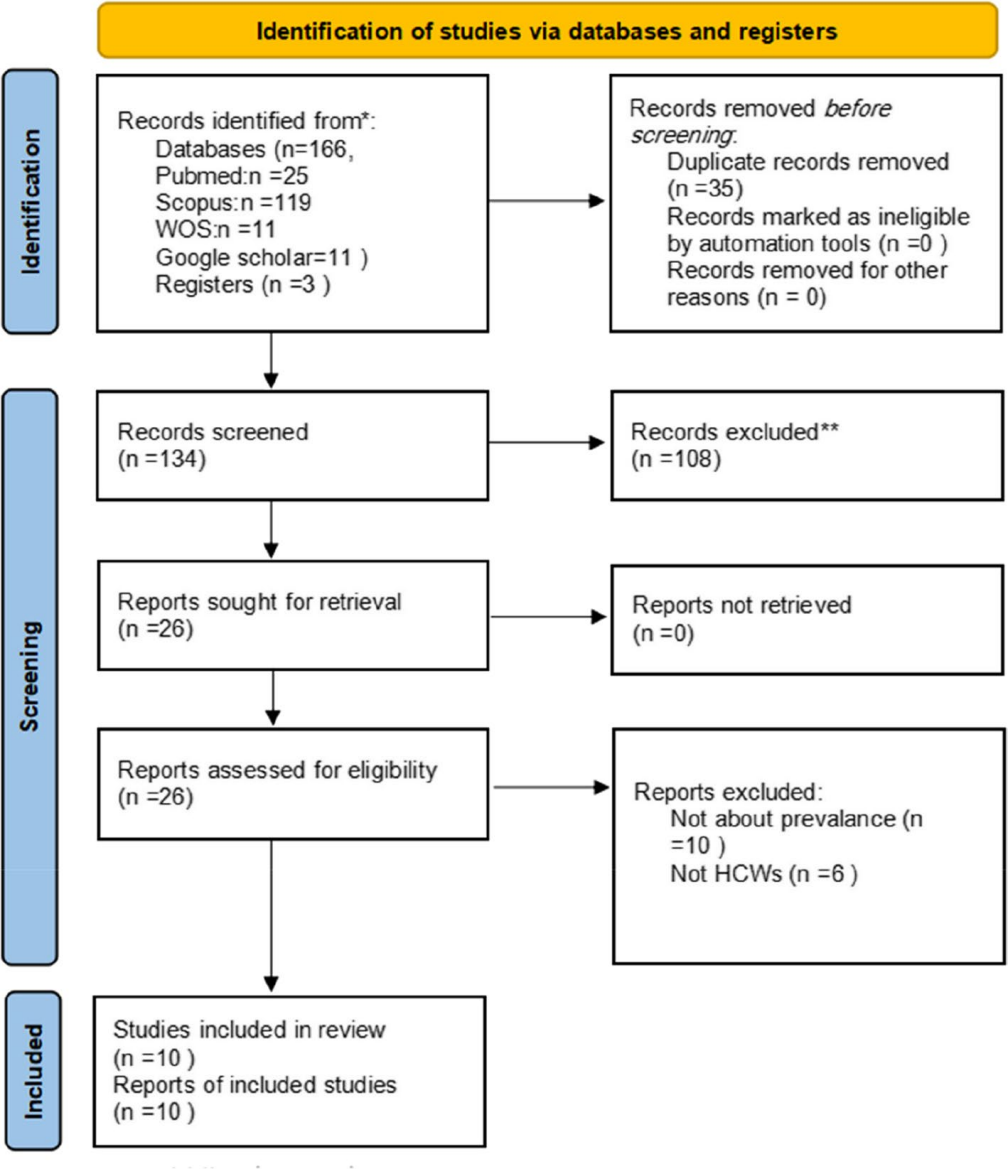
PRISMA flow chart of the search results

The initial database search resulted in 166 records from 4 databases: Scopus (*n* = 119), PubMed (*n* = 25), Google Scholar (*n* = 11), and Web of Science (*n* = 11). 21 records were included from the title and abstract screening which became eight after full-text screening and finally included ten studies in the data extraction (two studies were included from the manual screening of track citations). Figure 1 shows the screening stages via the PRISMA flow diagram.

### Characteristics of included articles

Among the included ten studies, eight were published in 2022 [17, 24–30], while two were published in 2020 [31] and 2023 [16]. The total number of included participants was 7322 subjects from 18 different countries across six WHO regions. All the studies [16, 17, 24–31] were cross-sectional and conducted across one country, except two were across two and 11 countries. The data collection dates of the included papers ranged from May 25, 2019, to December 28, 2022, with a span of several months for most studies. The sample sizes also varied widely, ranging from 75 to 3,856 participants. Similarly, the proportion of females among healthcare workers (HCWs) also varied significantly, ranging from 34.7% to 90.2%. The characteristics of included studies are in Table 2.

### Quality assessment

All the examined papers achieved high scores based on the criteria of the Newcastle–Ottawa Scale, with scores ranging from a minimum of 5 to a maximum of 10. Out of these papers, eight received a total score indicating good quality [16, 17, 25, 27–31], while one was rated as poor quality and another as fair quality. Table S2 in the Supplementary Materials illustrates the thorough point-by-point evaluation.

### Analysis of the outcomes

#### Proportion of mpox vaccination acceptance

**Fig. 2:**
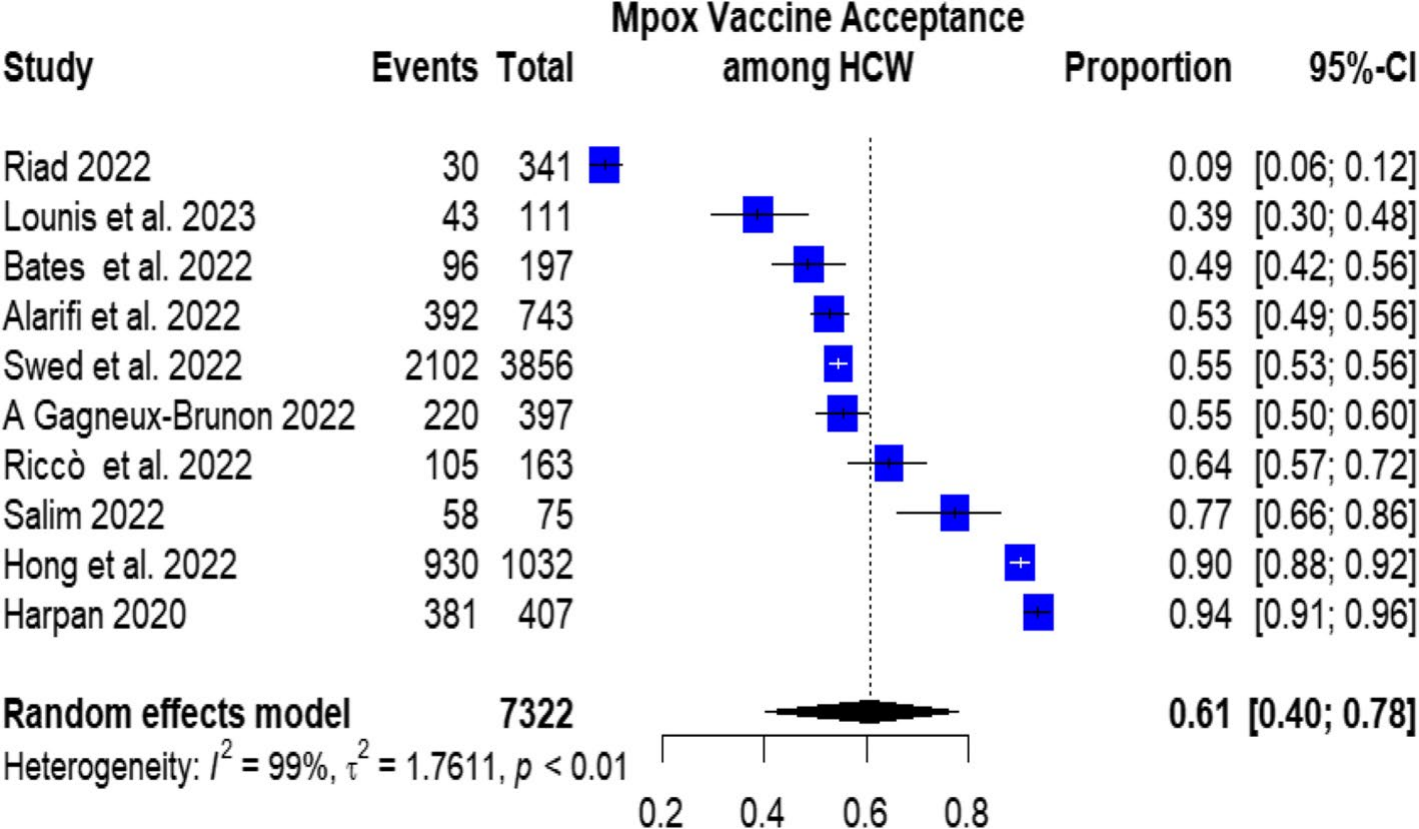
Mpox vaccine acceptance among HCW

The pooled prevalence of mpox vaccination acceptance among the included 7322 HCWs was 58.5% (95%CI: 40.5–67.4%), with significant heterogeneity among studies (I^2^ = 99.69%), as in Fig. 2.

#### Subgroup analysis of mpox vaccination acceptance by region

**Fig. 3:**
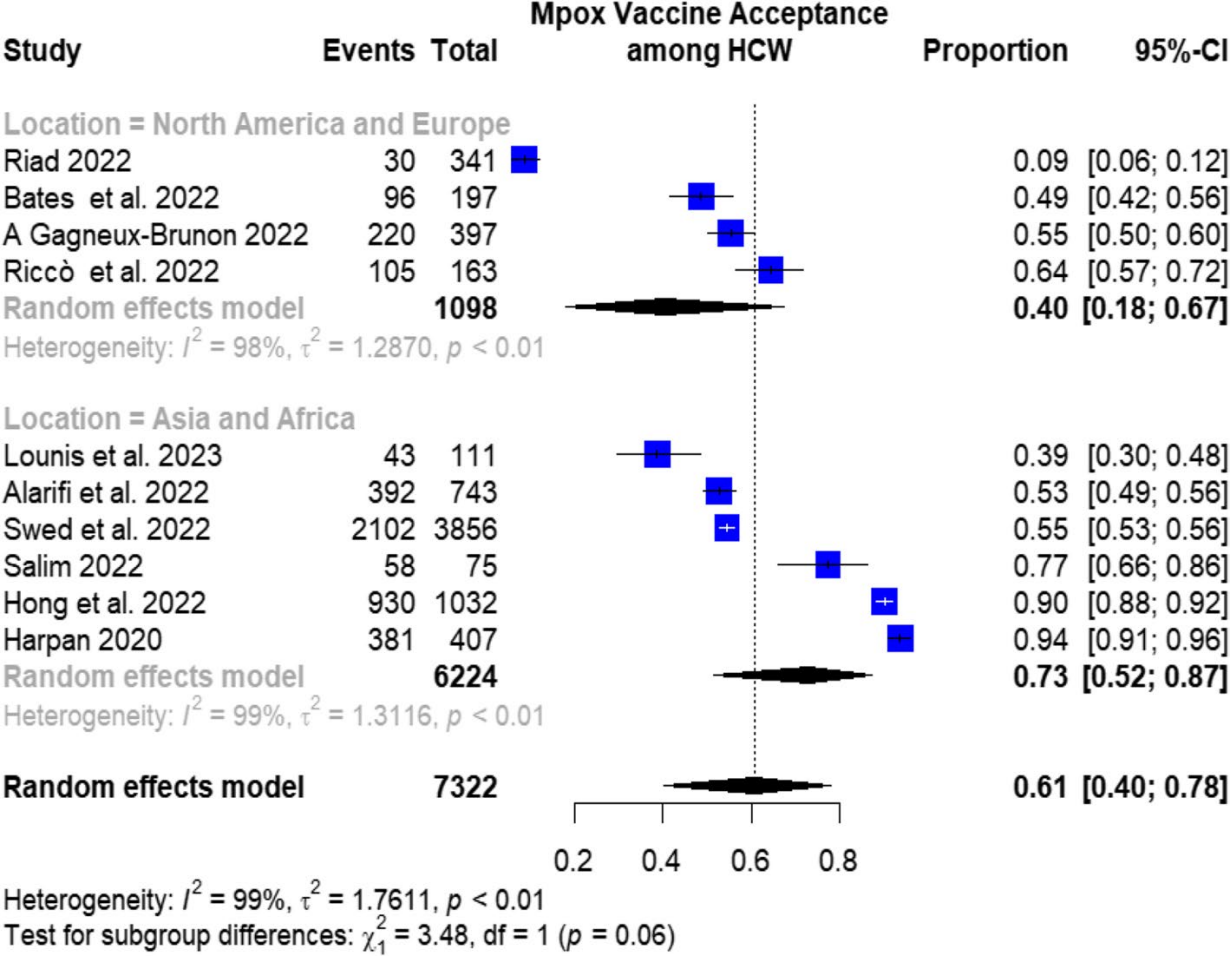
Subgroup analysis of mpox vaccination acceptance by regions

The subgroup analysis of mpox vaccination acceptance concerning different regions revealed a pooled prevalence of 44.3% (95%CI: 14.5–74%) in the countries located in North America and Europe continents. Meanwhile, the prevalence was found to be 68% (95%CI: 49.9–86.1%) in the countries located in Asian and African continents, as shown in Fig. 3.

#### Subgroup analysis of mpox vaccination acceptance regarding the target population

**Fig. 4:**
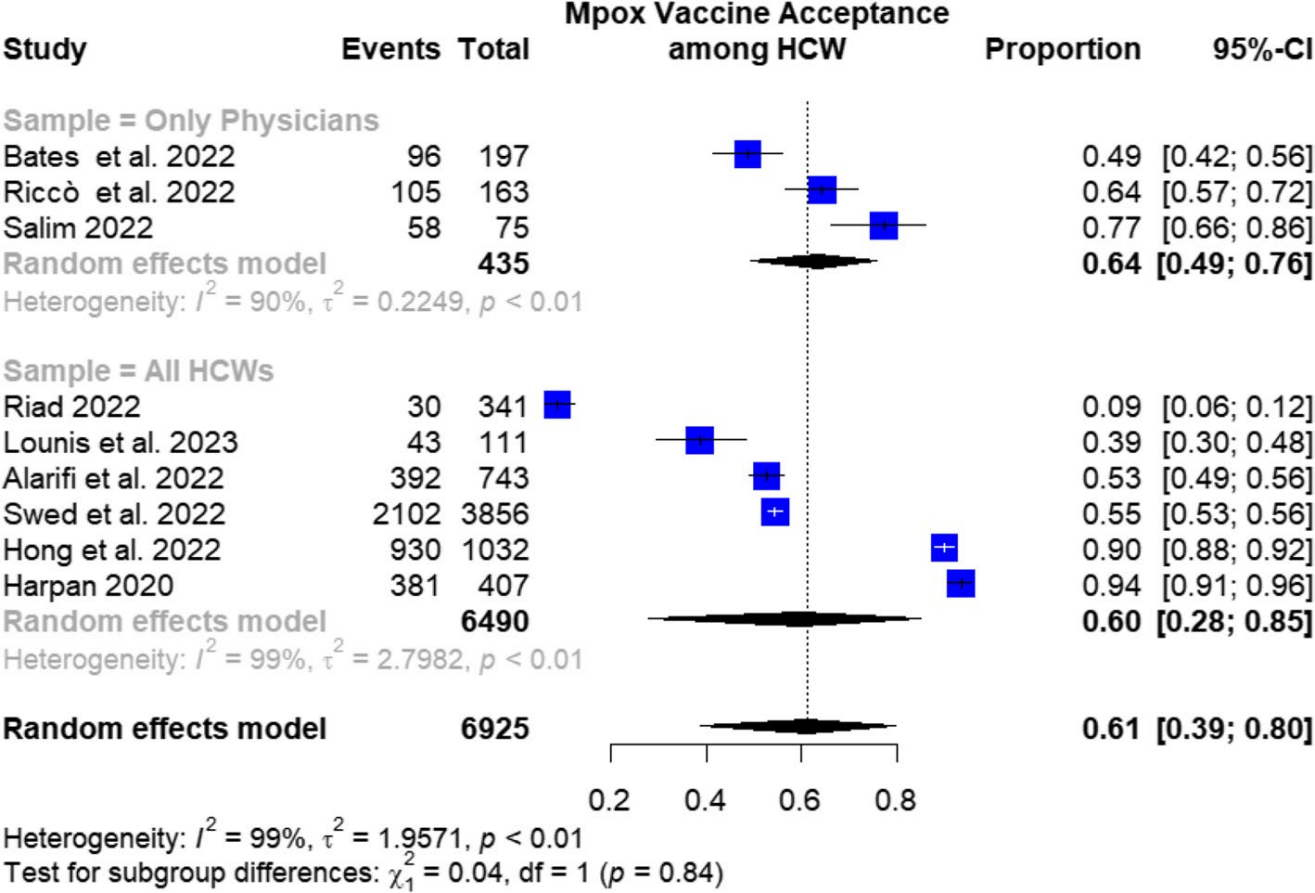
Subgroup analysis of mpox vaccination acceptance regarding the target population

In the subgroup analysis of mpox vaccination acceptance according to the target population of the included studies, the pooled prevalence was 77.1% (95%CI: 47.8–94.5%) in studies that exclusively enrolled physicians and 49% (95%CI: 21.7–76.3%) in those that included a sample of all healthcare workers, as shown in Fig. 4.

#### Proportion of mpox vaccination refusal

**Fig. 5:**
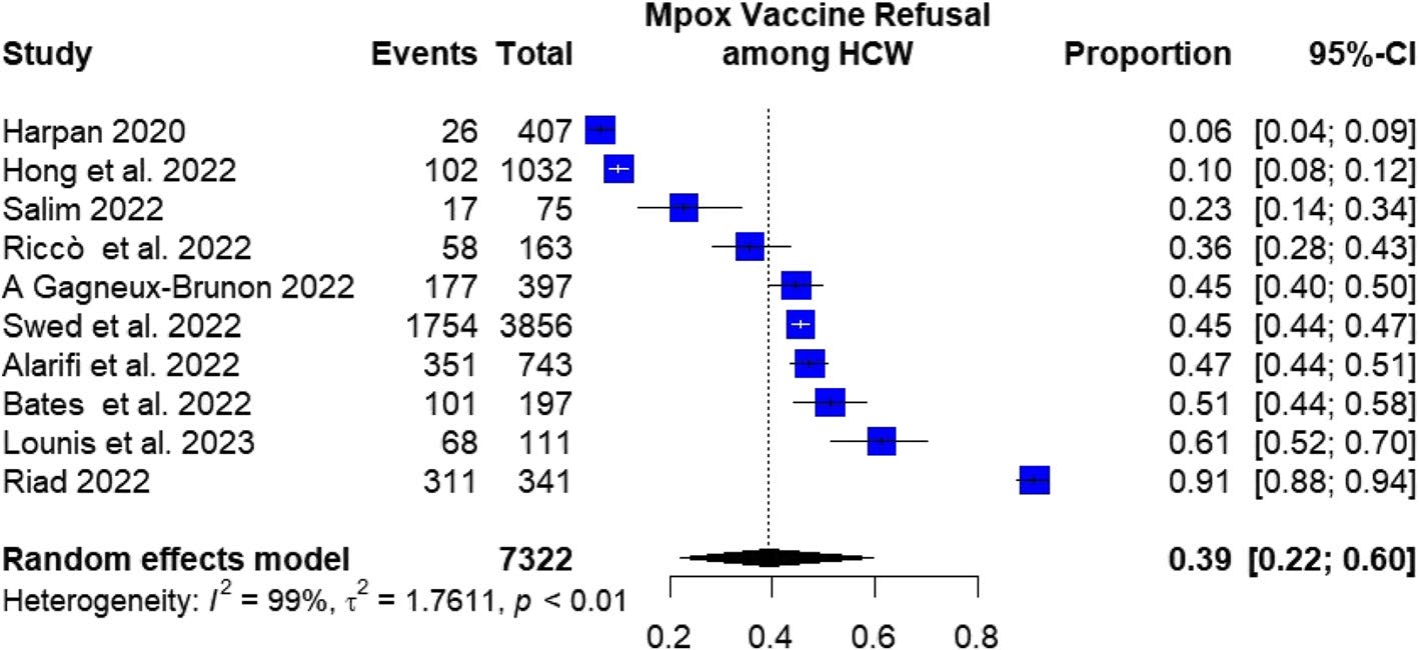
Mpox vaccine refusal among HCW

The pooled prevalence of mpox vaccination refusal among the included HCWs was 41.5% (95%CI: 23.6–59.5%), with significant heterogeneity among studies (I^2^ = 99.69%), as in Fig. 5.

#### Predictors of mpox vaccine uptake among healthcare workers

Ten studies investigated predictors of mpox vaccine uptake among HCWs [16, 17, 24–31]. Detailed results of the predictors are presented in Table 3 and S3. The authors investigated sociodemographic characteristics of the HCWs, mpox-related variables, and mpox vaccine-related variables as possible predictors of vaccine uptake among HCWs. mpox knowledge, social status, and getting influenza and covid-19 vaccine were positive predictors in studies, respectively. There was a wide range in values of odds and prevalence ratios among studies. For instance, HCWs who got COVID-19 vaccines were 2.5 to 5.4 times more likely to take one.

**Table 3:** Predictors of mpox vaccine acceptance among healthcare workers

Study ID	Predictors of mpox vaccine uptake among healthcare workers. (Increase/decrease/NS)							
	Chronic disease	Married	Experience duration	High monthly income	Physicians	Getting Covid-19 vaccine	Getting Influenza vaccine	Good HMPXV Perceived Knowledge
Bates et al. 2022 [29]	_	_	_	_	_	NS	NS	↑
Hong et al. 2022 [15]	_	_	_	_	_	_	_	_
Alarifi et al. 2022 [28]	_	_	↓	_	↑	↑	↑	↑
Swed et al. 2022 [27]	_	_	↓	_	_	_	_	_
Riccò et al. 2022 [25]	_	_	_	_	_	_	↑	NS
Harpan 2020 [23]	_	_	↑	↑	_	_	_	NS
Salim 2022 [24]	_	_	_	_	_	_	_	_
Riad 2022 [17]	NS	↓	_	_	NS	NS	↑	_
A Gagneux-Brunon 2022 [26]	_	_	_	_	NS	↑	_	_
Lounis et al. 2023 [30]	_	NS	NS	_	NS	↑	NS	NS

*NS* non-significant. ↑ more likely to accept. ↓ less likely to accept.—not investigated

### Publication bias

**Fig. 6:**
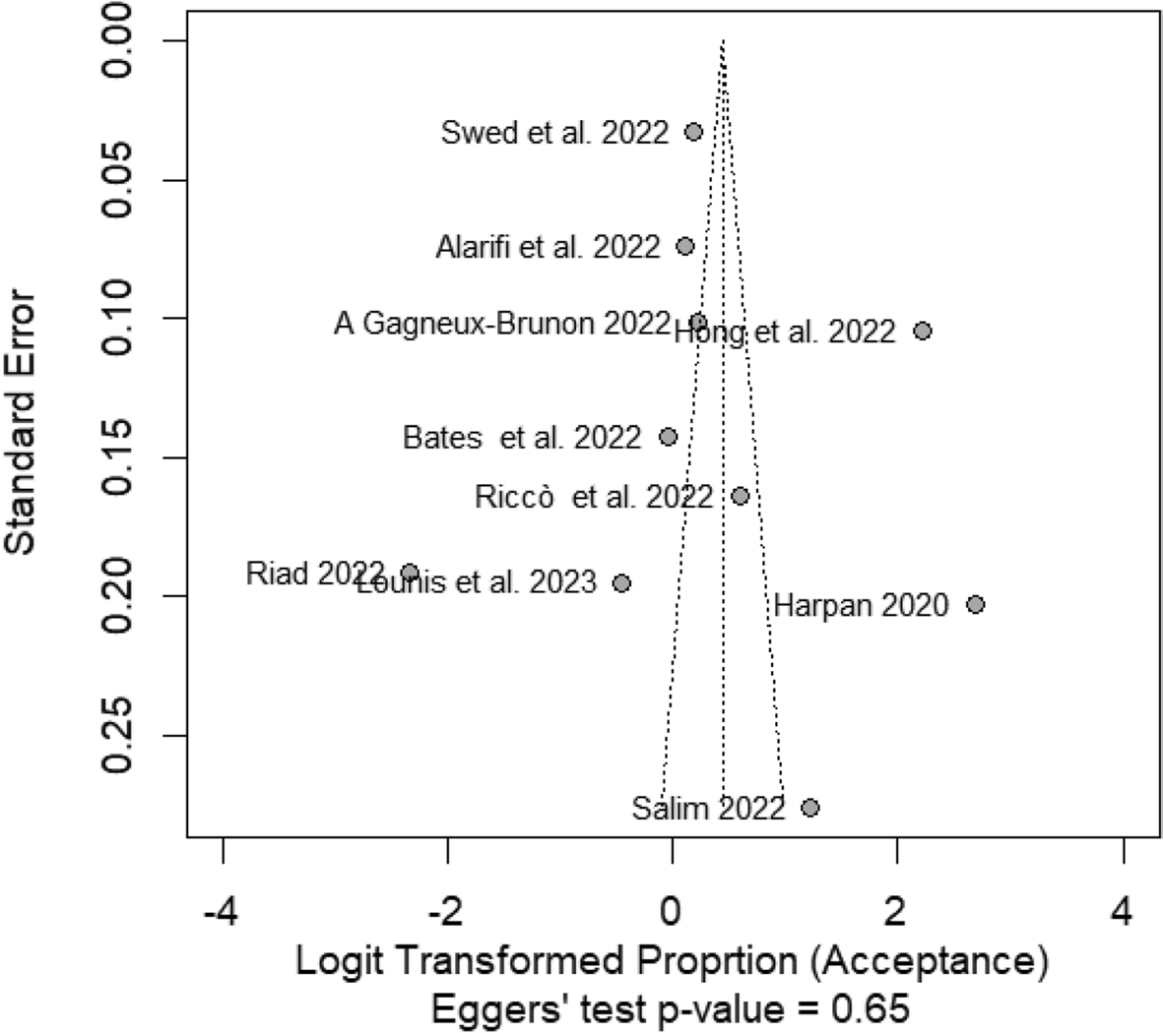
Publication bias was evaluated by Egger’s analysis

The Funnel plot to detect the publication bias in the included studies is summarized in Fig. 6. There was no evidence of publication bias in this meta-analysis (Egger’s test, *p* = 0.65).

## Discussion

Vaccination is generally the best method to prevent most diseases for which vaccines are currently available. It is not only essential to develop effective and safe vaccines but also to ensure fair distribution, proper logistical issues, and acceptance of the population to obtain the necessary demand from them [32]. Our findings indicate that the prevalence of mpox vaccine acceptance was 58.5%, and the prevalence of mpox vaccine refusal was 41.5%. There was a higher prevalence of acceptance in countries located in Asian and African areas compared to those in North America and Europe, estimated at 68% and 44.3%, respectively. Among the studies conducted solely among physicians, there was a high prevalence of mpox vaccine acceptance, at 77.1%, compared to 49% in studies that included all healthcare workers..

The higher propensity of vaccine acceptance in Asian and African continents (68%) may be attributable to higher knowledge of mpox in Asian countries, which is potentially a consequence of the Chinese government’s skillful propaganda efforts about mpox knowledge [33–35]. In addition, assessment of knowledge, awareness and practice of monkeypox among clinicians reveals poorer attitudes towards adopting preventive practices and an overall lower knowledge of monkeypox in the US as compared to China [29, 36]. Data regarding vaccinating attitudes with COVID-19 can be extrapolated to attitudes regarding mpox vaccination acceptance, and a similar pattern is observed in terms of geographic subgrouping with the sole exception of the Americas. HCWs in the region of the Americas had the highest in acceptance (70%), Asian and African Regions trailed behind with 67% and 57% acceptance. Whilst Europe HCWs had an overall 59% acceptance [37]. However, the pooled overall prevalence of acceptance rate was lower than the acceptance rate in four previous national cross-sectional studies with more than 2,000 participants (76.4% [4], 77.3% [24], and 90.12% [16], and 96.0% [31] respectively, slightly higher than the result in two national studies with more than 1000 participants, which indicated an overall acceptance rate of 52.7% [28], and 55.4% [26] respectively, and higher than the results of a multinational study with 3,856 participants, which indicated an acceptance rate of 54.5% [27]. The overall acceptance rate was affected by the small sample effect, in addition to adding the attitude of hesitation to the questionnaires, which also affected the overall acceptance rate. However, this result was significantly higher than the pooled acceptance rate of the mpox vaccine among the general population worldwide (65%), reported in a meta-analysis [38]. It is worth noting that the included studies lacked representation from Latin America, indicating a gap in research on mpox and its vaccines in that region [39–42].

Several racial, local, cultural, religious, and several other aspects may influence people’s perception of their acceptance of vaccination, in addition to the misinformation, as was observed during the COVID-19 pandemic and the refusal of the population to receive COVID-19 vaccination [43–46]. Given the dynamic nature of vaccine attitudes, challenges, and levels of misinformation and trust, continuous evaluation and close monitoring are essential for improving vaccine distribution and compliance. It is crucial for all countries, particularly those heavily affected by the mpox [47]outbreak, to prioritize implementing strategies that address misinformation and vaccine refusal [48]. Vaccination policies are implemented based on the available results, so providing the population with information based on preliminary evidence by all understandable and available means and forms is necessary. Therefore, the public health authorities must increase their use of social media to provide the population who needs vaccination with basic and worthy information to reduce the risk of mpox infection, especially in higher-risk groups, because social media is a vital resource and is almost the most essential tool in disease prevention nowadays.

The more the mpox outbreak develops, the greater the need for studies examining the effectiveness of mpox vaccines to increase awareness of their actual impact. Meanwhile, a recent study in the Netherlands found that among those non-primed vaccinated individuals with a 2-shot immunization series with the modified vaccine virus Ankara-Bavaria Nordic (MVA-BN, also known as Jynneos, Imvanex, or Imvamune) has partially low titers of mpox- neutralizing antibodies. Although the dose-sparing of the MVA-based influenza vaccine results in a low level of mpox-neutralizing antibodies, a third dose can significantly increase antibody immune response. Authors suggested follow-up studies on vaccinated individuals to evaluate the vaccine’s protective effect in high-risk groups due to the importance of mpox- neutralizing antibodies as a possible protection mechanism for the disease is not defined accurately [49].

Identifying specific populations with a lower rate of mpox vaccination intention will help public health authorities to develop more effective vaccination policies, as occurred with the COVID-19 pandemic [50]. Our study results constitute input towards the implemented measures for global vaccination against mpox among healthcare workers. Further research is needed to study factors associated with low mpox vaccination acceptance.

Our systematic review has limitations, mainly the high heterogeneity observed in the meta-analyses conducted. This heterogeneity cannot be fully explained through subgroup and sensitivity analyses. Likely, the variations in the surveyed populations and the different measurement instruments used across the included studies contribute to this heterogeneity. Further research is needed to understand better the factors influencing mpox vaccine acceptance and refusal among healthcare workers (HCWs) in diverse settings and populations. Additionally, it is essential to note that the number of studies from North America and Africa was limited. Therefore, conducting more research in these continents is necessary to comprehensively understand the mpox vaccine acceptance and refusal among HCWs. Furthermore, additional research is needed to investigate the role of healthcare workers (HCWs) in the overall vaccination strategy, examining the balance between vaccinating all HCWs and prioritizing those at high risk. Furthermore, additional research is needed to investigate the role of healthcare workers (HCWs) in the overall vaccination strategy, examining the balance between vaccinating all HCWs and prioritizing those at high risk. Acknowledging and addressing these limitations through future research will contribute to a more robust understanding of the topic and help inform targeted interventions and policies to improve vaccine acceptance and reduce refusal among HCWs in different regions.

## Conclusions

The prevalence of vaccination from healthcare workers is still lower than expected. Public health authorities could use these results for developing, designing, or promoting vaccination policies focused on these vulnerable and at-risk populations. Immunoprevention remains an essential public health intervention to prevent disease and probably transmission, even in mpox.

### Supplementary Information

**Additional file 1.** 

## Data Availability

Data available within the article or its supplementary materials.

## References

[1] Is pandemic finally over? We asked the experts. – Harvard Gazette. https://news.harvard.edu/gazette/story/2022/10/is-pandemic-finally-over-we-asked-the-experts/ (Accessed 7 May 2023).

[2] Emergencies: International health regulations and emergency committees. https://www.who.int/news-room/questions-and-answers/item/emergencies-international-health-regulations-and-emergency-committees (Accessed 7 May 2023).

[3] Sah R, et al. Public health emergency of international concern declared by the World Health Organization for Monkeypox. 2022; 7(1): 51–56 10.1080/23779497.2022.2124185.

[4] Alakunle E, Moens U, Nchinda G, and Okeke MI. Monkeypox virus in Nigeria: infection biology, epidemiology, and evolution. Viruses.2020; 12(11):1257 10.3390/V12111257. 10.3390/v12111257 PMC7694534 33167496

[5] Petersen E (2019). Human monkeypox: epidemiologic and clinical characteristics, diagnosis, and prevention. Infect Dis Clin North Am.

[6] Swed S (2023). Monkeypox post-COVID-19: knowledge, worrying, and vaccine adoption in the Arabic general population. Vaccines.

[7] Monkeypox. https://www.who.int/news-room/fact-sheets/detail/monkeypox (Accessed 11 Aug. 2022).

[8] Sah R, et al. The emergence of monkeypox: a global health threat. Cureus. 2022; 14(9): 10.7759/CUREUS.29304. 10.7759/cureus.29304 PMC9579419 36277578

[9] Multi-country outbreak of mpox, External situation report#27 – 14 August 2023. https://www.who.int/publications/m/item/multi-country-outbreak-of-mpox--external-situation-report-27---14-august-2023 (Accessed 24 Aug. 2023).

[10] Shchelkunov SN (2001). Human monkeypox and smallpox viruses: genomic comparison. FEBS Lett.

[11] Benites-Zapata VA (2022). Clinical features, hospitalisation and deaths associated with monkeypox: a systematic review and meta-analysis. Ann Clin Microbiol Antimicrob.

[12] Chakraborty C, Bhattacharya M, Ranjan Sharma A and Dhama K. Monkeypox virus vaccine evolution and global preparedness for vaccination. Int Immunopharmacol. 2022; 113:109346 10.1016/J.INTIMP.2022.109346. 10.1016/j.intimp.2022.109346 PMC9582788 36274490

[13] Monkeypox update: FDA authorizes emergency use of JYNNEOS vaccine to increase vaccine supply | FDA.” https://www.fda.gov/news-events/press-announcements/monkeypox-update-fda-authorizes-emergency-use-jynneos-vaccine-increase-vaccine-supply (Accessed 7 May 2023).

[14] Interim clinical considerations for use of JYNNEOS and ACAM2000 vaccines during the 2022 U.S. Mpox outbreak | Mpox | Poxvirus | CDC.” https://www.cdc.gov/poxvirus/mpox/clinicians/vaccines/vaccine-considerations.html?CDC_AA_refVal=https%3A%2F%2Fwww.cdc.gov%2Fpoxvirus%2Fmonkeypox%2Fclinicians%2Fsmallpox-vaccine.html (Accessed 7 May 2023).

[15] Sah R, et al. FDA’s authorized ‘JYNNEOS’ vaccine for counteracting monkeypox global public health emergency; an update – Correspondence. Int J Surg. 2022;107: 10697110.1016/J.IJSU.2022.106971. 10.1016/j.ijsu.2022.106971 PMC9617681 36330988

[16] Hong J, et al. The willingness of Chinese healthcare workers to receive monkeypox vaccine and its independent predictors: a cross-sectional survey. J Med Virol. 2023;95(1)e28294;10.1002/JMV.28294. 10.1002/jmv.28294 36367155

[17] Riad A (2022). Monkeypox knowledge and vaccine hesitancy of Czech healthcare workers: a Health Belief Model (HBM)-based study. Vaccines.

[18] Sahin TK, Erul E, Aksun MS, Sonmezer MC, Unal S, Akova M. Knowledge and attitudes of Turkish physicians towards human monkeypox disease and related vaccination: a cross-sectional study. Vaccines. 2022; 11(1):19 10.3390/VACCINES11010019/S1. 10.3390/vaccines11010019 PMC9863926 36679864

[19] Ajman F, et al. Healthcare workers’ worries and monkeypox vaccine advocacy during the first month of the WHO monkeypox alert: cross-sectional survey in Saudi Arabia. Vaccines.2022; 10(10):1408 10.3390/VACCINES10091408. 10.3390/vaccines10091408 PMC9503291 36146486

[20] Rethlefsen ML (2021). PRISMA-S: an extension to the PRISMA statement for reporting literature searches in systematic reviews. Syst Rev.

[21] Moskalewicz A, Oremus M (2020). No clear choice between Newcastle-Ottawa scale and appraisal tool for cross-sectional studies to assess methodological quality in cross-sectional studies of health-related quality of life and breast cancer. J Clin Epidemiol.

[22] R-4.3.0 for Windows. The R-project for statistical computing. https://cran.rstudio.com/bin/windows/base/index.html (Accessed 7 May 2023).

[23] Egger M, Smith GD, Schneider M, Minder C (1997). Bias in meta-analysis detected by a simple, graphical test. BMJ.

[24] Salim NA, Septadina IS, Permata M and Hudari H. Knowledge, attitude, and perception of anticipating 2022 global human monkeypox infection among internal medicine residents at Palembang Indonesia: an online survey. J Kedokt dan Kesehat Publ Ilm Fak Kedokt Univ Sriwij. 2022; 9(3):253–262 10.32539/JKK.V9I3.18799.

[25] Riccò M (2022). When a neglected tropical disease goes global: knowledge, attitudes and practices of Italian physicians towards monkeypox, preliminary results. Trop Med Infect Dis.

[26] Gagneux-Brunon A, Dauby N, Launay O, Botelho-Nevers E (2022). Attitudes towards monkeypox vaccination among healthcare workers in France and Belgium: an element of complacency?. J Hosp Infect.

[27] Swed S (2022). A multinational cross-sectional study on the awareness and concerns of healthcare providers toward monkeypox and the promotion of the monkeypox vaccination. SSRN Electron J.

[28] Alarifi AM, Alshahrani NZ and Sah R. Are Saudi Healthcare Workers (HCW) willing to receive the monkeypox virus vaccine? 2022; 10.20944/PREPRINTS202212.0562.V1. 10.3390/tropicalmed8080396 PMC10459197 37624334

[29] Bates BR, Grijalva MJ (2022). Knowledge, attitudes, and practices towards monkeypox during the 2022 outbreak: An online cross-sectional survey among clinicians in Ohio, USA. J Infect Public Health.

[30] Lounis M, Bencherit D, Abdelhadi S (2023). Knowledge and awareness of Algerian healthcare workers about human monkeypox and their attitude toward its vaccination: an online cross-sectional survey. Vacunas.

[31] Harapan H (2020). Acceptance and willingness to pay for a hypothetical vaccine against monkeypox viral infection among frontline physicians: a cross-sectional study in Indonesia. Vaccine.

[32] How to Prevent the Next Pandemic - Bill Gates - Google Books. https://books.google.com.eg/books?hl=en&lr&id=JA1SEAAAQBAJ&oi=fnd&pg=PT6&dq=Gates+B.+How+to+prevent+the+next+pandemic:+Knopf;+2022&ots=98JexAMphN&sig=C9jklrxjx0PMOQE1utpuxxqGw4c&redir_esc=y&pli=1#v=onepage&q=Gates%20B.%20How%20to%20prevent%20the%20next%20pandemic%3A%20Knopf%3B%202022&f=false. Accessed 7 May 2023.

[33] Alshahrani NZ (2022). Assessment of knowledge of monkeypox viral infection among the general population in Saudi Arabia. Pathogens.

[34] Sallam M, et al. Knowledge of human monkeypox and its relation to conspiracy beliefs among students in Jordanian Health Schools: filling the knowledge gap on emerging Zoonotic Viruses. Med. 2022; 58(7):924 10.3390/MEDICINA58070924. 10.3390/medicina58070924 PMC9317638 35888642

[35] Dong C, Yu Z, Zhao Y, Ma X. Knowledge and vaccination intention of monkeypox in China’s general population: A cross-sectional online survey. Travel Med Infect Dis. 2023;52;10253310.1016/J.TMAID.2022.102533. 10.1016/j.tmaid.2022.102533 PMC9759477 36543284

[36] Ren F, et al. Public awareness, specific knowledge, and worry about mpox (monkeypox): a preliminary community-based study in Shenzhen, China. Front Public Heal. 2023;11:1077564. 10.3389/FPUBH.2023.1077564/BIBTEX. 10.3389/fpubh.2023.1077564 PMC9971966 36866102

[37] Politis M, Sotiriou S, Doxani C, Stefanidis I, Zintzaras E, Rachiotis G (2023). Healthcare workers’ attitudes towards mandatory COVID-19 vaccination: a systematic review and meta-analysis. Vaccines.

[38] Ulloque-Badaracco JR (2022). Acceptance towards monkeypox vaccination: a systematic review and meta-analysis. Pathogens.

[39] Rodríguez-Morales AJ, Ortiz-Martínez Y and Bonilla-Aldana DK. What has been researched about monkeypox? a bibliometric analysis of an old zoonotic virus causing global concern. New Microbes New Infect. 2022; 47 10.1016/J.NMNI.2022.100993. 10.1016/j.nmni.2022.100993 PMC9243150 35782632

[40] Haider N (2022). Increased outbreaks of monkeypox highlight gaps in actual disease burden in Sub-Saharan Africa and in animal reservoirs. Int J Infect Dis.

[41] Al-Musa A, Chou J, LaBere B. The resurgence of a neglected orthopoxvirus: Immunologic and clinical aspects of monkeypox virus infections over the past six decades. Clin Immunol. 2022;243;10910810.1016/J.CLIM.2022.109108. 10.1016/j.clim.2022.109108 PMC9628774 36067982

[42] WHO update 79: Monkeypox outbreak update: situation - transmission - countermeasures. https://www.who.int/publications/m/item/update-79-monkeypox-outbreak-update (Accessed 7 May 2023).

[43] Guagliardo SAJ (2020). Asymptomatic orthopoxvirus circulation in humans in the wake of a monkeypox outbreak among chimpanzees in Cameroon. Am J Trop Med Hyg.

[44] de Albuquerque TR, Macedo LFR, de Oliveira EG, Neto MLR, de Menezes IRA (2022). Vaccination for COVID-19 in children: Denialism or misinformation?. J Pediatr Nurs.

[45] de Saint Laurent C, Murphy G, Hegarty K and Greene CM. Measuring the effects of misinformation exposure and beliefs on behavioural intentions: a COVID-19 vaccination study. Cogn Res Princ Implic. 7(1): 1–19 10.1186/S41235-022-00437-Y/FIGURES/6. 10.1186/s41235-022-00437-y PMC9526535 36183027

[46] Ganie AUR, Mukhter I (2022). Misinformation induced anxieties and fear affecting vaccination programs: Challenge for COVID-19 vaccination program. J Fam Med Prim Care.

[47] Rodriguez-Morales AJ, Franco OH. Public trust, misinformation and COVID-19 vaccination willingness in Latin America and the Caribbean: today’s key challenges. Lancet Reg Heal - Am. 2021;3;10007310.1016/j.lana.2021.100073. 10.1016/j.lana.2021.100073 PMC8432286 34522914

[48] Smith MJ, Marshall GS (2010). Navigating parental vaccine hesitancy. Pediatr Ann.

[49] Zaeck LM, et al. Low levels of monkeypox virus-neutralizing antibodies after MVA-BN vaccination in healthy individuals. Nat Med. 2022; 29(1):270–278 10.1038/s41591-022-02090-w. 10.1038/s41591-022-02090-w PMC9873555 36257333

[50] Alarcón-Braga EA, et al. Acceptance towards COVID-19 vaccination in Latin America and the Caribbean: a systematic review and meta-analysis. Travel Med Infect Dis. 2022;49:10236910.1016/J.TMAID.2022.102369. 10.1016/j.tmaid.2022.102369 PMC9169427 35680058

